# **MicroRNA signatures associated with radioiodine refractoriness and tumor dedifferentiation in metastatic papillary thyroid carcinoma**

**DOI:** 10.1007/s12020-025-04510-6

**Published:** 2026-03-09

**Authors:** Ana Kober Leite, Kelly Cristina Saito, Thérèse Rachell Theodoro, Fátima Solange Pasini, Venâncio Avancini Ferreira Alves, Luiz Paulo Kowalski, Maria Aparecida Silva Pinhal, Gilberto Mendes Junior Menderico, Edna Teruko Kimura, Leandro Luongo Matos

**Affiliations:** 1 Head and Neck Surgery Department, Instituto do Câncer do Estado de São Paulo, Hospital das Clínicas da Faculdade de Medicina da Universidade de São Paulo, São Paulo, SP Brazil; 2 Faculdade Israelita de Ciências da Saúde Albert Einstein, Hospital Albert Einstein, São Paulo, SP Brazil; 3 Department of Cell and Developmental Biology, Institute of Biomedical Sciences, University of São Paulo, São Paulo, SP Brazil; 4 Public Health Department, Faculdade de Medicina do ABC, São Paulo, SP Brazil; 5 Center for Translational Research in Oncology (LIM24), Instituto do Câncer do Estado de São Paulo Paulo (ICESP), Hospital das Clínicas (HCFMUSP), Faculdade de Medicina da Universidade de São Paulo, Sao Paulo, SP Brazil; 6 Comprehensive Center for Precision Oncology, Universidade de São Paulo (USP), São Paulo, Brazil; 7 Pathology Department, Laboratório de Investigação Médica 14 (LIM14), Instituto do Câncer do Estado de São Paulo, Faculdade de Medicina da Universidade de São Paulo, São Paulo, SP Brazil; 8 Biochemistry Department, Faculdade de Medicina do ABC, São Paulo, SP Brazil; 9 Head and Neck Surgery Department, Hospital das Clínicas da Faculdade de Medicina da Universidade de São Paulo, São Paulo, SP Brazil

**Keywords:** Papillary thyroid carcinoma, Prognosis, Metastasis, Molecular biology, MicroRNAs

## Abstract

**Purpose:**

Radioiodine (RAI) refractoriness and tumor dedifferentiation are major contributors to poor outcomes in metastatic papillary thyroid carcinoma (PTC). microRNAs (miRNAs) are promising biomarkers to identify these aggressive phenotypes. This study aimed to characterize miRNAs expression profiles associated with RAI refractoriness and dedifferentiation.

**Methods:**

From a cohort of 108 metastatic PTC patients, 24 cases with available formalin-fixed paraffin-embedded tissue were selected for molecular analysis, including 17 patients who died from disease progression and 7 long-term survivors with stable disease. Total RNA was extracted and analyzed by qPCR. miRNAs with differential expression (*P* < 0.10) in univariate analysis were included in multivariate linear regression models. Diagnostic performance was assessed using ROC curve analysis and hierarchical clustering.

**Results:**

The cohort had a mean age of 55.2 years and was predominantly male (66.7%). RAI-refractory disease was present in 63.2% of evaluable cases, and dedifferentiation in 33.3%. In multivariate analysis, lower expression of miR-200c-3p was independently associated with RAI refractoriness (*P* = 0.005). For tumor dedifferentiation, higher expression of let-7i-5p (*P* < 0.001) and miR-19b-3p (*P* < 0.001), and lower expression of miR-31-5p (*P* = 0.004) and let-7e-5p (*P* = 0.036), were independently associated. ROC curve analysis demonstrated high diagnostic accuracy for miR-200c-3p (AUC = 0.929) and let-7i-5p (AUC = 0.958) for, respectively, RAI refractoriness and tumor dedifferentiation. Heatmap analysis revealed clear segregation of phenotypic groups based on miRNA expression.

**Conclusions:**

Distinct miRNAs signatures are associated with RAI refractoriness and tumor dedifferentiation in metastatic PTC. These findings support their potential as prognostic biomarkers and may guide future therapeutic stratification.

**Supplementary Information:**

The online version contains supplementary material available at 10.1007/s12020-025-04510-6.

## Introduction

Papillary thyroid carcinoma (PTC) is the most common histological subtype of thyroid cancer and is generally associated with an excellent long-term prognosis [1]. However, a small proportion of patients develops distant metastases [2, 3], most frequently to the lungs and bones, which significantly worsens clinical outcomes [4]. Within this subset, two factors stand out as critical predictors of poor prognosis: radioiodine (RAI) refractoriness and tumor dedifferentiation. RAI-refractory tumors lose the ability to concentrate iodine, rendering adjuvant therapy ineffective, while dedifferentiation reflects a loss of epithelial features, often accompanied by increased invasiveness and resistance to conventional treatments. Both phenomena are strongly associated with disease progression and cancer-specific mortality [5, 6].

microRNAs (miRNAs) are small non-coding RNAs that regulate gene expression at the post-transcriptional level and play key roles in tumor biology [7, 8]. In thyroid cancer, deregulated miRNA expression has been linked to mechanisms of tumor initiation, progression, metastatic spread, and treatment resistance [9]. Certain miRNAs have been associated with the loss of iodine avidity and with aggressive histological phenotypes, including poorly differentiated and anaplastic variants [10]. Given their stability in biological samples and their functional relevance, miRNAs have emerged as promising biomarkers for identifying patients at higher risk of unfavorable outcomes, including therapeutic failure and disease-specific death [11]. Investigating these molecular signatures may contribute to the refinement of risk stratification models and the development of individualized treatment strategies.

RAI refractoriness and tumor dedifferentiation are well-established predictors of poor prognosis in PTC, particularly in patients with distant metastases. These phenotypes reflect underlying molecular alterations that compromise treatment efficacy and are strongly associated with disease progression and mortality. Identifying reliable biomarkers capable of detecting these aggressive disease patterns remains a clinical challenge. Among potential candidates, miRNAs have emerged as promising tools due to their involvement in key regulatory pathways and their stability in biological samples.

Recent studies have explored the relationship between microRNA expression and RAI refractoriness in PTC, although the topic remains under investigation. Pecce et al. analyzed 24 patients (14 RAI-refractory) and identified 15 dysregulated microRNAs, with particular emphasis on miR-139-5p, whose suppression was associated with lower intracellular iodine levels, whereas its overexpression restored iodine uptake and membrane localization of related transport proteins [12]. In contrast, Colombo et al. evaluated 39 PTCs and found no significant differences in microRNA profiles between RAI-avid and RAI-refractory tumors; however, expression patterns correlated with BRAF V600E status and molecular subtype (BRAF-like vs. RAS-like) [13]. More recently, Zhang et al. used integrated analyses of clinical samples and experimental models harboring the BRAF V600E mutation to identify miR-31 as a regulator of dedifferentiation and RAI resistance. Functional assays demonstrated that miR-31 overexpression suppressed thyroid differentiation genes and sodium–iodide symporter (NIS) activity, while its inhibition restored NIS expression and RAI uptake, implicating miR-31 in the loss of RAI avidity in aggressive PTCs [14].

In this context, the present study aimed to characterize miRNA expression profiles associated with RAI refractoriness and tumor dedifferentiation in patients with metastatic PTC.

## Methods

### Ethics

The study was approved by the Institutional Review Board (IRB; CAAE 44997215.1.0000.0065). For patients who were alive and under follow-up at the institution’s outpatient clinic, informed consent was obtained during routine clinical visits. For deceased patients or those who could not be reached to provide consent, the IRB granted a waiver of informed consent.

## Data collection

Among all patients treated for papillary thyroid carcinoma (PTC) by the Head and Neck Surgery Department of the Instituto do Câncer do Estado de São Paulo, Hospital das Clínicas, Faculdade de Medicina da Universidade de São Paulo (ICESP, HCFMUSP), a total of 108 individuals were identified as having distant metastases (DM). Patients in this cohort were either managed entirely within the institution or referred after undergoing initial surgical intervention at other facilities. The cohort comprised cases diagnosed and surgically treated between 1986 and 2015, during which a total of 3,555 PTC patients underwent surgical treatment at our institution.

From the original cohort of 108 patients with metastatic papillary thyroid carcinoma, a subset of 24 cases was selected for microRNA analysis. The inclusion criterion for this subset was the availability of primary tumor formalin-fixed paraffin-embedded (FFPE) material of sufficient quality to allow reliable molecular extraction and downstream profiling.

Pathological staging was reviewed and updated according to the 8th edition of the AJCC/UICC Cancer Staging Manual [15]. Patients were included if they had a confirmed diagnosis of DM either at initial staging or during follow-up.

The diagnosis of DM was established based on clinical evaluation and/or positive findings on Iodine-131 whole-body scintigraphy (WBS), chest X-ray, computed tomography (CT), magnetic resonance imaging (MRI), or tissue biopsy. As per institutional protocol, all patients underwent chest radiography preoperatively, with CT reserved for selected cases. The presence of radiological or pathological evidence of distant disease at diagnosis was sufficient to classify a case as metastatic. Currently, WBS is routinely performed only in patients eligible for radioactive iodine (RAI) therapy or in those with suspected persistent or recurrent disease. In certain cases, biopsy of metastatic lesions was performed to confirm diagnosis, particularly when the primary site was uncertain, or the clinical presentation was atypical.

Dedifferentiation was defined histologically on the primary tumor or metastatic lesion. It was considered present when a well-differentiated PTC underwent abrupt transition to a high-grade morphology lacking the characteristic histological features of the original tumor. In cases presenting dedifferentiation in the primary tumor, the differentiated area was selected for analysis.

Iodine avidity was assessed by visual uptake at known metastatic sites on the post-therapeutic WBS following the first adjuvant RAI administration under thyroid hormone stimulation. Tumors were classified as radioiodine-refractory according to the 2015 American Thyroid Association guidelines, which define refractoriness as follows: absence of RAI uptake in metastatic sites; loss of RAI uptake after previous evidence of avidity; heterogeneous RAI uptake across lesions; or disease progression despite RAI concentration in metastatic foci [16]. Only patients who received RAI therapy were considered for the definition RAI-refractoriness.

Patients were classified as having died from PTC if they exhibited symptomatic, extensive, and progressive disease at the last follow-up, and if this was listed as the main or a contributing cause of death on official death certificates.

## Selection of samples for molecular study

Tumor samples used for molecular analysis were obtained from FFPE tissue blocks archived following routine histopathological examination. All patients who died from papillary thyroid carcinoma and had representative tumor tissue available in FFPE form at the institution were initially eligible. From this group, cases were selected for molecular testing. A control group of metastatic patients with long-term follow-up and stable disease was matched at a 2:1 ratio. This resulted in the inclusion of 16 patients who died due to disease progression and 8 controls with non-progressive metastatic disease, totaling 24 cases. However, during the course of the analysis, one additional control patient died from distant metastatic disease. This patient was reclassified, yielding a final cohort of 17 deceased patients and 7 long-term survivors.

## Molecular assays

### MicroRNA detection technique

miRNA expression was quantified by reverse transcription–quantitative polymerase chain reaction (qPCR) according to a previously established protocol [17]. Total RNA was extracted from ten 5-µm sections of formalin-fixed paraffin-embedded tissue using the MagMAX FFPE RNA Ultra Kit (Applied Biosystems, Foster City, CA, USA), following the manufacturer’s instructions. Reverse transcription with polyadenylation was performed to generate cDNA, which was subsequently amplified using the TaqMan Low Density Array (TLDA) platform (Thermo Fisher Scientific^®^, Waltham, MA, USA). The TLDA system plate accommodates 64 commercially available microRNAs in triplicate, and the panel was customized by the authors based on literature review. Specifically, microRNAs were selected according to prior reports in papillary thyroid carcinoma, their association with aggressive tumor behavior in other malignancies, and additional candidates considered biologically relevant in the current literature. All reactions were conducted in triplicate, and cycle threshold (CT) values were obtained using the Thermo Fisher Scientific^®^ cloud-based analysis platform.

MicroRNAs with CT values > 36 were considered undetectable and excluded. Furthermore, only miRNAs detected in at least 70% of the samples were retained for statistical analysis. Based on these criteria, the following 18 microRNAs were excluded from the final dataset: miR-129-5p, miR-130b-3p, miR-137-3p, miR-138-2-3p, miR-146b-3p, miR-155-3p, miR-17-3p, miR-187-3p, miR-302c-3p, miR-30e-5p, miR-34b-3p, miR-34c-5p, miR-455-3p, miR-4788, miR-506-3p, miR-654-3p, miR-9-5p, and miR-98-5p.

Expression data were normalized across samples using the quantile normalization method implemented in the Expander software platform [18]. MicroRNAs with detectable expression in 100% of the samples (let-7 g-5p, miR-125a-5p, miR-181a-5p, miR-200a-3p, and miR-200b-3p) were evaluated for stability using the NormFinder algorithm [19]. The two most stable (let-7 g-5p and miR-181a-5p) were selected, and their geometric mean was used as an endogenous control, as previously described by Vandesompele et al. [20] and applied in the group’s prior studies [11, 17]. Relative expression levels of each microRNA were calculated using the 2^–ΔCt^ method to determine upregulation or downregulation. The complete list of primers used in the qPCR analysis is provided in Supplementary Table S2, with target microRNAs selected based on a comprehensive literature review to encompass key pathways involved in tumor progression and aggressiveness.

### Statistical analyzes

Statistical analyses were performed using SPSS software, version 29.0 (IBM^®^, Endicott, NY, USA). Continuous variables were described using means and standard deviation (SD) or standard error (SE), while categorical variables were summarized as absolute and relative frequencies. Group comparisons for continuous variables were conducted using the Mann–Whitney U test. Cut-off values for quantitative variables were determined by receiver operating characteristic (ROC) curve analysis, and categorical variables were evaluated through direct association of categories. Variables with a P value < 0.10 in univariate analysis were included in multivariate models, which were constructed using linear regression to identify independent associations with the outcomes of interest. Diagnostic performance of selected microRNAs was assessed by ROC curve analysis, with estimation of area under the curve (AUC), sensitivity, and specificity. Expression values were standardized using Z-scores, and negative Z-scores were applied for downregulated microRNAs. Heatmaps and hierarchical clustering were generated using MultiExperiment Viewer (MeV), version 4.5.0 (available at: http://mev.tm4.org).

## Results

The descriptive characteristics of the 24 patients with metastatic papillary thyroid carcinoma included in the study are summarized in Table 1. Most patients were male (66.7%), with a mean age of 55.2 ± 13.1 years at diagnosis. Multifocality (54.2%), extrathyroidal extension (69.6%), and vascular invasion (71.4%) were frequently observed histopathological features.

**Table 1:** Descriptive data of patients with metastatic papillary thyroid carcinoma included in the study

Variable	Total *n* = 24 (%)	
**Sex**		
Female	8 (33.3%)	
Male	16 (66.7%)	
**Age**		
Mean ± SD (years-old)	55.2 ± 13.1	
**Histopathological data**		
Extrathyroidal extension *(missing: 1)*	16 (69.6%)	
Vascular invasion *(missing: 3)*	15 (71.4%)	
Multifocality	13 (54.2%)	
Size (mean ± SD; cm)	4.5 ± 3.3 cm	
**Initial pT classification (AJCC 8th edition;** *missing: 1***)**		
pT1	5 (21.7%)	
pT2	3 (13%)	
pT3	7 (30.4%)	
pT4a	4 (17.4%)	
pT4b	4 (17.4%)	
**Initial pN classification (AJCC 8th edition;** *missing: 1***)**		
pN0	10 (43.5%)	
pN1a	4 (17.4%)	
pN1b	9 (39.1%)	
**Treatment**		
Total thyroidectomy	23 (95.8%)	
Central compartment neck dissection	14 (58.4%)	
Level II-V neck dissection	10 (41.7%)	
R0 surgery *(missing: 2)*	19 (86.4%)	
Radioiodine therapy (RIT)	19 (79.2%)	
**Number of RIT**		
One	10 (52.6%)	
> 2	9 (47.4%)	
**Distant metastases**		
Lung	23 (95.8%)	
Liver	4 (16.7%)	
Bones	13 (54.2%)	
Multiple sites	15 (62.5%)	
Diagnosis with the primary tumor	16 (66.7%)	
Dedifferentiation	8 (33.3%)	
**Follow-up**		
Radioiodine refractory disease (valid: 19*)	12 (63.2%)	
Nodal recurrence *(missing: 3)*	8 (33.3%)	
Cancer-related death	17 (70.8%)	
Follow-up time (mean ± SD; months)	83 ± 61.2 (median: 60.5)	

*Note: Radioiodine refractory disease was considered only amongst patients that received RIT

Nearly all patients underwent total thyroidectomy (95.8%), and most received radioiodine therapy (79.2%), with more than one dose administered in 47.4% of cases. The lungs were the most common site of distant metastasis (95.8%), followed by bone (54.2%) and liver (16.7%). Notably, 62.5% of patients presented with metastases to multiple organs. Tumor dedifferentiation occurred in one-third of the patients (33.3%), and radioiodine-refractory disease was observed in 63.2% of those with available data.

The median follow-up time was 60.5 months (mean ± SD: 83 ± 61.2), during which 70.8% of patients died due to disease progression. Regional recurrence occurred in 33.3% of cases.

The microRNA expression profile associated with radioiodine refractoriness and tumor dedifferentiation is summarized in Table 2. Only patients who received RAI therapy (*n* = 19) were considered for the analysis of RAI-refractoriness. In the univariate analysis (Mann-Whitney U test), miR-200c-3p was significantly down expressed in patients with radioiodine-refractory disease (*P* = 0.003), while miR-21-5p (*P* = 0.083) and miR-31-5p (*P* = 0.093) showed a trend toward higher expression in this group. In relation to tumor dedifferentiation, several microRNAs were differentially expressed. let-7i-5p (*P* = 0.015), miR-125a-5p (*P* = 0.045) and miR-19a-3p (*P* = 0.017) were significantly overexpressed and miR-31-5p (*P* = 0.019) was down expressed in dedifferentiated tumors. Additionally, let-7e-5p (*p* = 0.080), miR-19b-3p (*p* = 0.056) showed trends toward differential expression in the same context.

**Table 2:** MicroRNA expression profile associated with radioiodine refractoriness and tumor dedifferentiation in metastatic papillary thyroid carcinoma

miR	RADIOIODINE-REFRACTORY				Exact *P*-value^+^	DEDIFFERENTIATION				Exact *P*-value^+^
	YES (*n* = 12)		NO (*n* = 7)			YES (*n* = 8)		NO (*n* = 16)		
	MEAN	SE	MEAN	SE		MEAN	SE	MEAN	SE	
let-7b-5p	0.075	0.046	0.068	0.039	0.432	0.069	0.030	0.085	0.037	0.871
let-7c-5p	3.368	1.065	4.365	1.587	0.635	8.265	6.942	4.146	0.943	0.274
let-7d-5p	0.411	0.046	0.454	0.096	0.967	0.425	0.079	0.466	0.048	0.452
let-7e-5p	3.500	1.223	5.072	2.389	0.596	1.662	0.400	4.679	1.352	0.080*
let-7f-5p	3.354	0.407	6.158	1.854	0.482	2.917	0.441	4.804	0.878	0.214
let-7i-5p	15.411	3.829	16.181	3.042	0.432	28.128	5.972	13.236	1.669	0.015**
miR-1-3p	13.041	3.869	17.230	12.909	0.482	15.768	11.968	16.531	5.992	0.261
miR-101-3p	2.611	0.654	2.754	0.923	0.592	4.085	0.801	2.319	0.538	0.123
miR-10b-5p	0.473	0.153	0.687	0.285	0.724	0.443	0.144	0.605	0.167	0.891
miR-125a-5p	6.867	1.146	8.209	3.446	0.733	4.257	0.621	8.800	1.625	0.045**
miR-138-5p	3.184	0.948	2.178	0.780	0.660	3.365	1.568	3.120	0.740	1.000
miR-141-3p	14.052	3.614	17.966	4.722	0.211	14.071	3.778	14.973	3.283	1.000
miR-146b-5p	3.451	0.920	4.137	2.050	0.733	1.702	0.522	4.264	1.111	0.241
miR-16-5p	4.043	1.172	5.136	3.144	0.837	3.933	1.041	4.399	1.558	0.417
miR-17-5p	1.146	0.239	1.799	0.996	0.813	2.028	0.782	1.061	0.261	0.238
miR-181b-5p	0.304	0.060	0.428	0.078	0.285	0.303	0.048	0.345	0.061	0.570
miR-18a-5p	0.067	0.058	0.032	0.030	0.570	0.068	0.033	0.074	0.053	0.147
miR-191-5p	0.704	0.142	0.598	0.278	0.315	0.873	0.190	0.752	0.211	0.365
miR-199a-3p	0.979	0.228	1.630	1.242	0.432	0.873	0.340	1.304	0.532	0.653
miR-19a-3p	0.284	0.095	0.628	0.586	0.310	0.890	0.429	0.180	0.056	0.017**
miR-19b-3p	0.514	0.166	1.480	1.009	0.859	3.650	2.203	0.483	0.143	0.056*
miR-200a-3p	3.312	0.540	3.451	1.185	0.733	2.985	0.882	3.544	0.601	0.569
miR-200b-3p	41.987	8.144	65.198	23.598	0.536	28.587	10.843	48.927	11.592	0.120
miR-200c-3p	14.719	2.716	39.228	6.608	0.003**	15.768	5.978	24.306	4.335	0.261
miR-203a-3p	0.090	0.042	0.034	0.020	0.562	0.177	0.079	0.042	0.018	0.210
miR-205-5p	1.879	0.866	1.107	0.512	0.913	5.625	2.784	1.211	0.431	0.553
miR-20a-5p	0.306	0.049	0.519	0.275	0.791	0.588	0.216	0.305	0.061	0.131
miR-21-5p	38.573	9.004	59.043	47.812	0.083*	27.161	10.446	53.530	20.952	0.264
miR-214-3p	0.320	0.134	1.989	1.931	0.604	0.425	0.171	1.136	0.754	1.000
miR-221-3p	23.547	7.302	22.987	6.741	0.650	18.989	6.819	23.628	5.644	0.922
miR-222-3p	1.899	0.506	1.560	0.329	0.902	0.994	0.216	1.985	0.392	0.198
miR-29a-3p	5.191	0.721	6.037	2.565	0.299	5.244	0.413	5.719	1.206	0.720
miR-30a-5p	3.155	0.705	6.576	3.235	0.364	4.260	0.569	5.006	1.717	0.391
miR-30b-5p	10.775	2.895	12.951	6.687	0.724	9.984	1.493	13.435	3.969	0.548
miR-30c-5p	16.435	5.879	20.814	10.088	0.930	21.099	4.987	18.423	6.458	0.332
miR-30d-5p	1.345	0.389	1.647	0.650	0.930	1.791	0.427	1.488	0.409	0.142
miR-30e-3p	0.504	0.128	0.842	0.323	0.428	0.622	0.110	0.668	0.176	0.701
miR-31-5p	2.683	2.299	1.510	0.463	0.093*	0.154	0.127	2.577	1.615	0.019**
miR-34a-5p	3.929	0.670	5.895	2.170	0.791	2.811	0.960	5.085	1.019	0.123
miR-423-5p	0.616	0.207	0.651	0.126	0.425	0.356	0.090	0.706	0.151	0.106
miR-429	0.366	0.130	0.165	0.038	0.525	0.290	0.069	0.310	0.106	0.535
miR-483-3p	0.905	0.683	0.242	0.064	0.713	2.047	1.904	0.695	0.454	0.736
miR-92a-3p	6.238	1.355	24.685	17.110	0.930	7.765	3.690	14.194	7.568	0.535

*Note*: SE = standard error; ^+^Mann-Whitney U test; *P < 0.10; **P < 0.05

miRNA with p-values lower than 0.1 were submitted to multivariate logistic regression analysis to identify those expressed independently between the two groups, as shown in Table 3. Patients with radioiodine-refractory disease exhibited significantly lower expression of miR-200c-3p, as demonstrated in the multivariate linear regression analysis (model 1). This microRNA was independently associated with refractoriness (B = − 0.019; *P* = 0.005), with a strong negative standardized coefficient, indicating that reduced expression is predictive of loss of iodine avidity. In the context of tumor dedifferentiation, four microRNAs were identified as independently associated with this phenotype. Higher expression levels of let-7i-5p (B = 0.017; *P* < 0.001) and miR-19b-3p (B = 0.566; *P* < 0.001) were associated with dedifferentiation, whereas miR-31-5p (B = − 0.020; *P* = 0.004) and let-7e-5p (B = 0.022; *P* = 0.036) was inversely associated, suggesting downregulation in dedifferentiated tumors.

**Table 3:** Multivariate linear regression identifying prognostic MicroRNAs related to radioiodine refractoriness and tumor dedifferentiation in metastatic papillary thyroid carcinoma

model		Unstandardized coefficients		Standardized coefficients	*P*-value	95% CI (for Beta)	
		B	SE	Beta		Lower	Upper
**RADIOIODINE-REFRACTORY**							
1*	(Constant)	1.064	0.164		< 0.001	0.712	1.416
	***miR-200c-3p***	**-0.019**	**0.006**	**-0.663**	0.005	-0.031	-0.007
**DEDIFFERENTIATION**							
1	(Constant)	-0.236	0.122		0.082	-0.508	0.036
	let-7i-5p	0.025	0.006	0.791	0.002	0.011	0.039
2	(Constant)	-0.326	0.087		0.005	-0.523	-0.130
	let-7i-5p	0.019	0.005	0.586	0.003	0.008	0.029
	miR-19b-3p	0.409	0.115	0,510	0.006	0.148	0.670
3	(Constant)	-0.280	0.064		0.002	-0.428	-0.132
	let-7i-5p	0.016	0.003	0.502	0.002	0.008	0.024
	miR-19b-3p	0.507	0.089	0.632	< 0.001	0.302	0.711
	miR-31-5p	-0.019	0.006	-0.313	0.015	-0.032	-0.005
4**	(Constant)	-0.394	0.066		0.001	-0.550	-0.238
	**let-7i-5p**	**0.017**	**0.003**	**0.549**	**< 0.001**	**0.011**	**0.024**
	**miR-19b-3p**	**0.566**	**0.072**	**0.706**	**< 0.001**	**0.397**	**0.736**
	**miR-31-5p**	**-0.020**	**0.005**	**-0.335**	**0.004**	**-0.031**	**-0.009**
	**let-7e-5p**	**0.022**	**0.009**	**0.213**	**0.036**	**0.002**	**0.042**

*Legend*: SE = standard-error

* miR-21-5p (B = 0.165; *P* = 0.196) and miR-31-5p (B = 0.136; *P* = 0.181) not included in model 1

** miR-125a-5p (B=-0.063; *P* = 0.495) and miR-19a-3p (B=-0.125; *P* = 0.678) not included in final model (model 4)

**Fig. 1:**
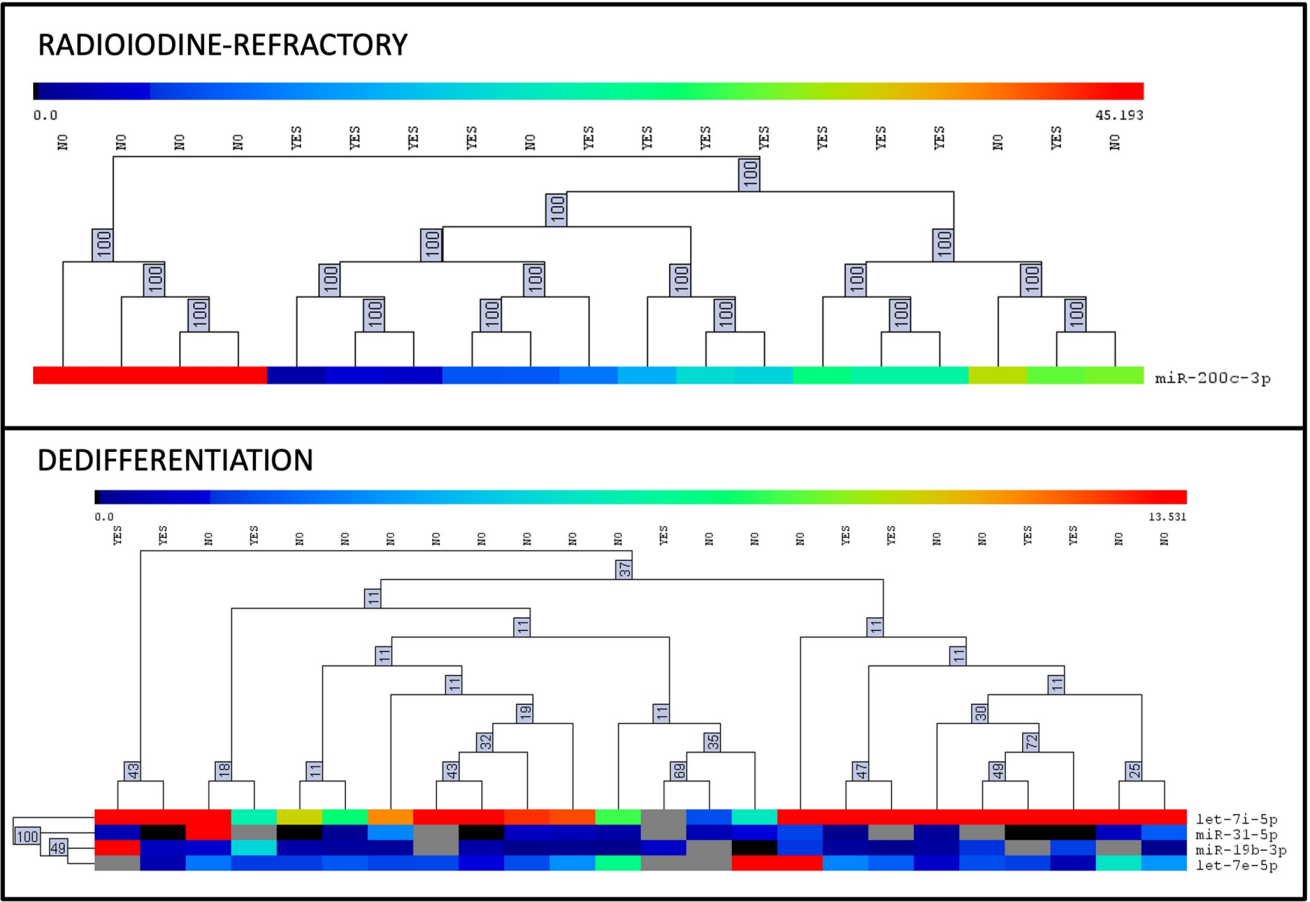
Heatmaps and hierarchical clustering of microRNAs identified in the multivariate linear regression analysis as prognostic markers of radioiodine refractoriness (top panel) and tumor dedifferentiation (bottom panel) in metastatic papillary thyroid carcinoma. Only microRNAs significantly associated with each phenotype are shown: miR-200c-3p for radioiodine refractoriness; let-7i-5p, miR-31-5p, miR-19b-3p, and let-7e-5p for tumor dedifferentiation. Yellow indicates high expression, and blue indicates low expression across tumor samples

Heatmap and hierarchical clustering analyses using the microRNAs identified in the multivariate linear regression models are shown in Fig. 1. For radioiodine-refractory disease, expression levels of miR-200c-3p alone were sufficient to distinguish between refractory and non-refractory cases, with clear segregation between the two groups based on expression intensity. For tumor dedifferentiation, the combined analysis of let-7i-5p, miR-19b-3p, miR-31-5p, and let-7e-5p resulted in robust separation between dedifferentiated and well-differentiated tumors.

**Fig. 2:**
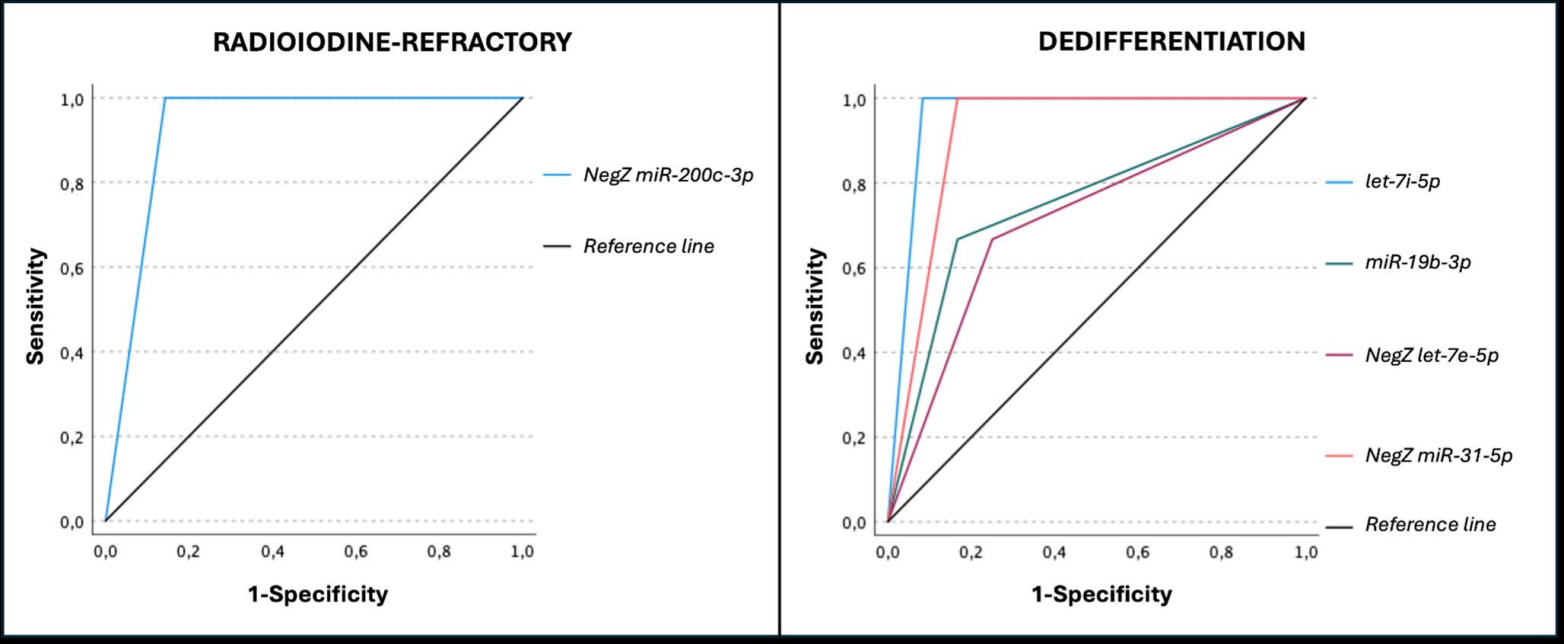
ROC curves demonstrating the discriminatory performance of selected prognostic microRNAs for radioiodine refractoriness (left panel) and tumor dedifferentiation (right panel) in metastatic papillary thyroid carcinoma. For radioiodine refractoriness, NegZ miR-200c-3p showed an AUC of 0.929. For tumor dedifferentiation, the AUCs were 0.958 for let-7i-5p, 0.917 for NegZ miR-31-5p, 0.750 for miR-19b-3p, and 0.708 for NegZ let-7e-5p. The diagonal black line represents the reference (non-discriminatory) line (AUC = 0.5) *Note*: “NegZ” indicates a negative Z-score (downregulated microRNA)

Cut-off values for the differently expressed microRNAs were established based on ROC curve analysis for the identification of radioiodine-refractory disease and tumor dedifferentiation in patients with metastatic papillary thyroid carcinoma. These microRNAs demonstrated high discriminatory performance, with AUCs ranging from 0.708 to 0.958, as shown in Table 4 and illustrated in Fig. 2. Strong individual performance of miR-200c-3p for radioiodine refractoriness (AUC = 0.929) and let-7i-5p for tumor dedifferentiation (AUC = 0.958) was estimated.

**Table 4:** Evaluation of diagnostic accuracy of MicroRNAs *200c-3p* for radioiodine refractoriness and *let7i-5p*, *19b-3p*, *31-5p* and *let-7e-5p* for tumor dedifferentiation in patients with metastatic papillary thyroid carcinoma

micro-RNA	Sensitivity	Specificity	AUC	95% CI (AUC)	
				Lower	upper
**RADIOIODINE-REFRACTORY**					
*NegZ miR-200c-3p* ≥ − 0.378	1.000	0.857	0.929	0.771	1.086
**DEDIFFERENTIATION**					
*let-7i-5p* ≥ 18.756	1.000	0.917	0.958	0.856	1.061
*miR-19b-3p* ≥ 0.657	0.667	0.833	0.750	0.404	1.096
*NegZ miR-31-5p* ≥ 0.316	1.000	0.833	0.917	0.771	1.062
*NegZ let-7e-5p* ≥ 0.452	0.667	0.750	0.708	0.357	1.059

*Legend*: “NegZ” = negative Z-score (downregulated microRNA); AUC = area under the ROC curve

## Discussion

**Fig. 3:**
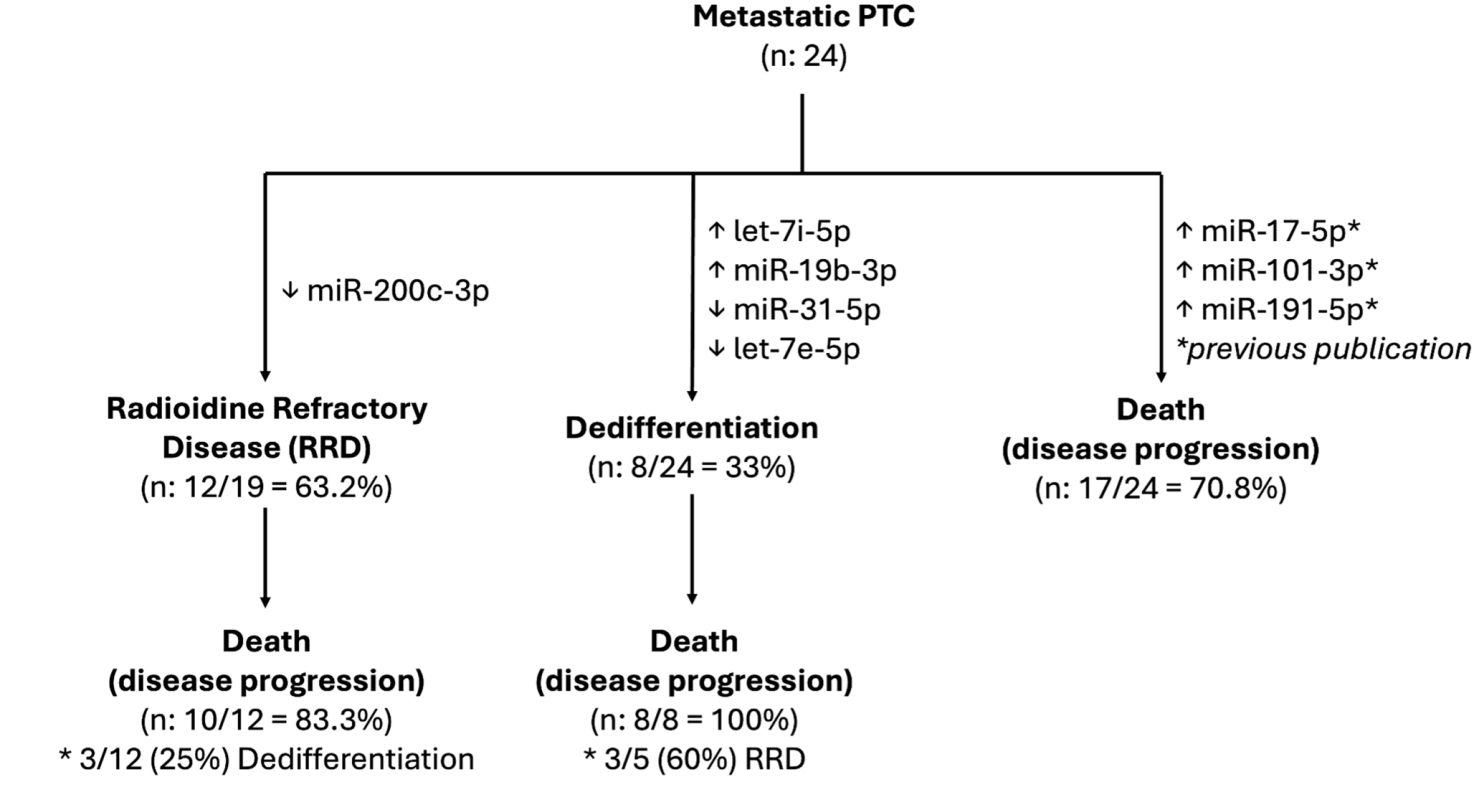
Schematic representation of the associations between differentially expressed microRNAs (miRs) and clinically relevant outcomes in patients with metastatic papillary thyroid carcinoma (PTC). Lower expression of miR-200c-3p was independently associated with radioiodine-refractory disease (RRD), which affected 63.2% of patients and was linked to a high rate of disease-specific mortality (83.3%). Tumor dedifferentiation occurred in 33% of the cohort and was associated with increased expression of let-7i-5p and miR-19b-3p, and decreased expression of miR-31-5p and let-7e-5p. Among dedifferentiated tumors, 100% of patients died from disease progression, and 60% also exhibited RRD. In a previous publication from our group [11], increased expression of miR-17-5p, miR-101-3p, and miR-191-5p was identified in patients who died due to metastatic progression

Although a few previous studies have evaluated the role of microRNAs (miRNAs) in papillary thyroid carcinoma (PTC), our study presents one of the most comprehensive assessments to date of differentially expressed miRNAs specifically associated with radioiodine refractoriness and tumor dedifferentiation in patients with metastatic disease. These two clinical features are key determinants of poor prognosis and remain poorly explored from a molecular standpoint, particularly in cohorts with well-defined long-term outcomes. In this study, miR-200c-3p was significantly underexpressed in RAI-refractory tumors, while let-7i-5p and miR-19b-3p were overexpressed, and miR-31-5p and let-7e-5p were underexpressed in dedifferentiated tumors. The ability of these miRNAs to accurately discriminate aggressive biological phenotypes—reflected in AUCs of up to 0.958—highlights their translational potential as biomarkers for clinical decision-making. In addition, previous work from our group [11] identified miR-17-5p, miR-101-3p, and miR-191-5p as significantly overexpressed in patients who died from disease progression, reinforcing the link between specific miR expression patterns and unfavorable outcomes. The integration of these findings is synthesized in Fig. 3, illustrating the association between distinct miRNA profiles and clinically relevant endpoints such as radioiodine refractoriness, dedifferentiation, and cancer-specific mortality.

miRNAs play a critical role in radioiodine-refractory PTC by modulating key molecular pathways involved in iodine uptake, tumor aggressiveness, and therapeutic resistance. In a study evaluating patients with non-radioiodine-avid lung metastases, 13 serum miRNAs were found to be significantly differentially expressed compared to those with iodine-avid metastases. Among them, five miRNAs were upregulated (miR-1249, miR-106a, miR-503, miR-34c-5p, and miR-1281) and eight were downregulated (miR-1915, miR-2861, miR-3196, miR-500, miR-572, miR-33b, miR-554, and miR-18a). Furthermore, bioinformatic analysis identified miR-106a as a central regulatory element within the miRNA–mRNA interaction network, modulating the expression of 193 target genes [21]. In our study, although the specific microRNAs differed, we similarly identified miR-200c-3p as significantly underexpressed in RAI-refractory metastatic PTC. This observation aligns mechanistically with prior evidence suggesting that downregulation of miR-200c promotes epithelial–mesenchymal transition (EMT), enhances tumor cell survival, and reduces iodine uptake—all of which contribute to therapeutic resistance. Previous studies have also shown that diminished miR-200c expression upregulates anti-apoptotic signaling pathways in thyroid cancer cells, thereby conferring a survival advantage and facilitating resistance to radioactive iodine therapy [22]. Our findings not only reinforce the biological relevance of miR-200c in the context of radioiodine refractoriness but also highlight its potential as a biomarker for the early identification of patients at increased risk of treatment failure.

Dedifferentiation plays a critical role in shaping both the prognosis and therapeutic approach in metastatic PTC. It refers to the process by which well-differentiated cancer cells progressively lose their specialized morphological and functional characteristics, acquiring a more primitive and less differentiated phenotype. This transition is associated with increased tumor aggressiveness and a marked reduction in responsiveness to conventional therapies, including radioactive iodine, ultimately contributing to poorer clinical outcomes [11, 23]. A study previously reported a markedly poor prognosis in patients with this biological profile, [24] which is further reflected in the present study by the 100% mortality rate among metastatic PTC patients exhibiting tumor dedifferentiation. Several microRNAs have been identified as differentially expressed in association with dedifferentiation in PTC, particularly in the metastatic setting. Notably, prior studies have described the downregulation of miR-204-5p, miR-135a-5p, and let-7f, along with the overexpression of other members of the let-7 family, suggesting complex regulatory roles of these miRNAs in maintaining differentiation and controlling tumor aggressiveness [9, 25–29]. Considering our specific findings, the overexpression of let-7i and miR-19b, along with the underexpression of miR-31 and let-7e in dedifferentiated metastatic PTC, aligns with previous reports associating these miRs with more aggressive tumor behavior. The elevated expression of let-7i and miR-19b has been linked to enhanced cellular proliferation and survival, potentially contributing to loss of differentiation and treatment resistance [26, 30]. These alterations may reflect a shift toward a more undifferentiated and malignant phenotype, reinforcing the role of specific miRNAs as molecular markers of biological aggressiveness in advanced PTC. Conversely, the reduced expression of miR-31 and let-7e has been associated with the loss of differentiated cellular features and increased invasiveness, underscoring their potential role in promoting metastatic progression and tumor dedifferentiation [14, 31–33] The interplay among these miRNAs offers important insights into the molecular mechanisms underlying PTC progression and highlights their potential utility as prognostic biomarkers and therapeutic targets.

The inclusion of miR-181a-5p in the normalization strategy warrants clarification. This microRNA was selected based on its stable expression across all samples and its combination with let-7 g-5p provided a robust normalization factor within this metastatic cohort. Although previous studies have reported variable expression patterns of miR-181a-5p in PTC, these findings were mostly derived from tumor-versus-normal comparisons and may not apply to cohorts composed exclusively of advanced or metastatic cases [34–36]. Nevertheless, the choice of reference microRNAs represents a potential limitation, as expression stability may differ across disease contexts.

The present study has additional limitations that should be acknowledged. First, the selection of cases was based on the availability of FFPE tumor samples, which may introduce selection bias. Second, the cohort included a disproportionately high rate of disease-specific mortality—exceeding that typically reported in the literature—reflecting the deliberate inclusion of patients with clinically aggressive disease for molecular analysis. This intentional enrichment likely contributed to the high frequency of radioiodine-refractory disease and tumor dedifferentiation observed in our sample. As a consequence, it was not feasible to investigate microRNAs differentially expressed in relation to mortality within each of these subgroups individually, due to the lack of outcome heterogeneity. Despite these limitations, it is important to emphasize that distant metastases, dedifferentiation, and cancer-related death are rare events in PTC, and the aggregation of such cases in a single, well-characterized cohort represents a unique strength of this study.

## Conclusion

This study identified distinct microRNA expression profiles that are significantly associated with radioiodine refractoriness and tumor dedifferentiation in metastatic papillary thyroid carcinoma, both of which are strongly linked to adverse prognosis. The underexpression of miR-200c-3p in RAI-refractory tumors, along with the differential expression of let-7i-5p, miR-19b-3p, miR-31-5p, and let-7e-5p in dedifferentiated tumors, suggest that these miRs may serve as valuable prognostic biomarkers. Their high discriminatory accuracy reinforces their potential role in improving risk stratification and guiding therapeutic decisions, particularly in advanced cases where conventional parameters are limited. These findings not only deepen our molecular understanding of aggressive PTC but also establish a foundation for future studies aimed at validating the clinical applicability of these biomarkers and exploring their potential as therapeutic targets.

## Supplementary Information

Below is the link to the electronic supplementary material.

Supplementary Material 1

## Data Availability

The data that support the findings of this study are available from the corresponding author, upon reasonable request.

## Declarations

During the preparation of this work, the author(s) used ChatGPT (OpenAI) to assist with language refinement, grammar correction, and formatting of scientific writing. After using this tool, the author(s) reviewed and edited the content as needed and take full responsibility for the content of the publication.
