# Supplementary material for: MicroRNA signatures associated with radioiodine refractoriness and tumor dedifferentiation in metastatic papillary thyroid carcinoma
Source: Endocrine. 2026 Mar 9;91(1):94. doi: 10.1007/s12020-025-04510-6 (PMC12971732; doi:10.1007/s12020-025-04510-6)
Supplement: Supplementary file 1 — Supplementary Material 1 [file 12020_2025_4510_MOESM1_ESM.docx]

**SUPPLEMENT**

**Table S1.** Primers sequences for the studied microRNAs.

| **microRNA** | **Sequence** |
| --- | --- |
| *let-7b-5p* | UGAGGUAGUAGGUUGUGGUU |
| *let-7c-5p* | UGAGGUAGUAGGUUGUAUGGUU |
| *let-7d-5p* | AGAGGUAGUAGGUUGCAUAGUU |
| *let-7e-5p* | UGAGGUAGGAGGUUGUAUAGUU |
| *let-7f-5p* | UGAGGUAGUAGAUUGUAUAGUU |
| *let-7g-5p* | UGAGGUAGUAGUUUGUACAGUU |
| *let-7i-5p* | UGAGGUAGUAGUUUGUGCUGUU |
| *miR-1-3p* | UGGAAUGUAAAGAAGUAUGUAU |
| *miR-101-3p* | UACAGUACUGUGAUAACUGAA |
| *miR-10b-5p* | UACCCUGUAGAACCGAAUUUGUG |
| *miR-125a-5p* | UCCUGAGACCCUUUAACCUGUGA |
| *miR-129-5p* | CUUUUUGCGGUCUGGGGCUUGC |
| *miR-130b-3p* | CAGUGCAAUGAUGAAAGGGCAU |
| *miR-137-3p* | UUAUUGCUUAAGAAUACGCGUAG |
| *miR-138-2-3p* | GCUAUUUCACGACACCAGGGUU |
| *miR-138-5p* | AGCUGGUGUUGUGAAUCAGGCCG |
| *miR-141-3p* | UAACACUGUCUGGGUAAAGAUGG |
| *miR-146b-3p* | UGCCCUGUGGACUCAGUUCUGG |
| *miR-146b-5p* | UGAGAACUGAAUUCCAUAGGCU |
| *miR-155-3p* | CUCCUACAUAUUAGCAUUAACA |
| *miR-16-5p* | UAGCAGCACGUAAAUAUUGGCG |
| *miR-17-3p* | ACUGCAGUGAAGCACUUGUAG |
| *miR-17-5p* | CAAAGUGCUUACAGUGCAGGUAG |
| *miR-181a-5p* | AACAUUCAACGCUGUCGGUGAGU |
| *miR-181b-5p* | AACAUUCAUUGCUGUCGGUGGGU |
| *miR-187-3p* | UCGUGUCUUGUGUGCAGCCGG |
| *miR-18a-5p* | UAAGGUGCAUCUAGUGCAGAUAG |
| *miR-191-5p* | CAACGGAAAUCCCAAAAGCAGCUG |
| *miR-199a-3p* | ACAGUAGUCUGCACAUUGGUUA |
| *miR-19a-3p* | UGUGCAAAUCUAUGCAAAACUGA |
| *miR-19b-3p* | UGUGCAAAUCCAUGCAAAACUGA |
| *miR-200a-3p* | UAACACUGUCUGGGUAACGAUGU |
| *miR-200b-3p* | UAAUACUGCCUGGUAAUGAUGA |
| *miR-200c-3p* | UAAUACUGCCGGGUAAUGAUGGA |
| *miR-203a-3p* | GUGAAAUGUUUAGGACCACUAG |
| *miR-205-5p* | UCCUUCAUUCCACCGGAGUCUG |
| *miR-20a-5p* | UAAAGUGCUUAUAAGUGCAGGUAG |
| *miR-21-5p* | UAGCUUAUCAGACUGAUGUUGA |
| *miR-214-3p* | ACAGCAGGCACAGACAGGCAGU |
| *miR-221-3p* | AGCUACAUUGUCUGCUGGGUUUC |
| *miR-222-3p* | AGCUACAUCUGGCUACUGGGU |
| *miR-29a-3p* | UAGCACCAUCUGAAAUCGGUUA |
| *miR-302c-3p* | UAAGUGCUUCCAUGUUUCAGUGG |
| *miR-30a-5p* | UGUAAACAUCCUCGACUGGAAG |
| *miR-30b-5p* | UGUAAAACAUCCUACACUCAGCU |
| *miR-30c-5p* | UGUAAACAUCCUACACUCUCAGC |
| *miR-30d-5p* | UGUAAACAUCCCCGACUGGAAG |
| *miR-30e-3p* | CUUUCAGUCGGAUGUUUACAGC |
| *miR-30e-5p* | UGUAAAACAUCCUUGACUGGAAG |
| *miR-31-5p* | AGGCAAGAUGCUGGCAUAGCU |
| *miR-34a-5p* | UGGCAGUGUCUUAGCUGGUUGU |
| *miR-34b-3p* | CAAUCACUAACUCCACUGCCAU |
| *miR-34c-5p* | AGGCAGUGUAGUAGCUGAUUGC |
| *miR-423-5p* | UGAGGGGCAGAGAGCGAGACUUU |
| *miR-429* | UAAUACUGUCUGGUAAAACCGU |
| *miR-455-3p* | GCAGUCCAUGGGCAUAUACAC |
| *miR-4788* | UUACGGACCAGCUAAGGGAGGC |
| *miR-483-3p* | UCACUCCUCUCCUCCCGUCUU |
| *miR-506-3p* | UAAGGCACCCUUCUGAGUAGA |
| *miR-654-3p* | UAUGUCUGCUGACCAUCACCUU |
| *miR-9-5p* | UCUUUGGUUAUCUAGCUGUAUGA |
| *miR-92a-3p* | UAUUGCACUUGUCCCGGCCUGU |
| *miR-98-5p* | UGAGGUAGUAAGUUGUAUUGUU |
